# The Blood-Brain Barrier Breakdown During Acute Phase of the Pilocarpine Model of Epilepsy Is Dynamic and Time-Dependent

**DOI:** 10.3389/fneur.2019.00382

**Published:** 2019-04-16

**Authors:** Natália Ferreira Mendes, Aline Priscila Pansani, Elis Regina Ferreira Carmanhães, Poliana Tange, Juliana Vieira Meireles, Mayara Ochikubo, Jair Ribeiro Chagas, Alexandre Valotta da Silva, Glaucia Monteiro de Castro, Luciana Le Sueur-Maluf

**Affiliations:** ^1^Departamento de Biociências, Universidade Federal de São Paulo, Santos, Brazil; ^2^Departamento de Neurologia e Neurocirurgia, Universidade Federal de São Paulo, São Paulo, Brazil; ^3^Departamento de Psicobiologia, Universidade Federal de São Paulo, São Paulo, Brazil

**Keywords:** epilepsy, blood-brain barrier, pilocarpine, status epilepticus, Evans blue, sodium fluorescein

## Abstract

The maintenance of blood-brain barrier (BBB) integrity is essential for providing a suitable environment for nervous tissue function. BBB disruption is involved in many central nervous system diseases, including epilepsy. Evidence demonstrates that BBB breakdown may induce epileptic seizures, and conversely, seizure-induced BBB disruption may cause further epileptic episodes. This study was conducted based on the premise that the impairment of brain tissue during the triggering event may determine the organization and functioning of the brain during epileptogenesis, and that BBB may have a key role in this process. Our purpose was to investigate in rats the relationship between pilocarpine-induced status epilepticus (SE), and BBB integrity by determining the time course of the BBB opening and its subsequent recovery during the acute phase of the pilocarpine model. BBB integrity was assessed by quantitative and morphological methods, using sodium fluorescein and Evans blue (EB) dyes as markers of the increased permeability to micromolecules and macromolecules, respectively. Different time-points of the pilocarpine model were analyzed: 30 min after pilocarpine injection and then 1, 5, and 24 h after the SE onset. Our results show that BBB breakdown is a dynamic phenomenon and time-dependent, i.e., it happens at specific time-points of the acute phase of pilocarpine model of epilepsy, recovering in part its integrity afterwards. Pilocarpine-induced changes on brain tissue initially increases the BBB permeability to micromolecules, and subsequently, around 5 h after SE, the BBB breakdown to macromolecules occurs. After BBB breakdown, EB dye is captured by damaged cells, especially neurons, astrocytes, and oligodendrocytes. Although the BBB permeability to macromolecules is restored 24 h after the start of SE, the leakage of micromolecules persists and the consequences of BBB degradation are widely disseminated in the brain. Our findings reveal the existence of a temporal window of BBB dysfunction in the acute phase of the pilocarpine model that is important for the development of therapeutic strategies that could prevent the epileptogenesis.

## Introduction

The blood-brain barrier (BBB) is a physical and functional interface between blood and brain, essential for providing a suitable environment for neuronal function, regardless of fluctuations in blood composition (1–3). Many of the BBB properties are dependent on a close association among brain capillaries and astrocytes, which contain multiple processes that extend toward neurons and vessels (4, 5). The end-feet of the astrocytes surround about 95% of the abluminal surface of the brain capillaries (6) and are responsible for releasing several factors that induce and maintain the BBB phenotype (7). Besides astrocytes, multiple agents, and cell types including pericytes, neurons, and perivascular microglia are involved in the modulation of BBB permeability. These cells interact with microvessels, regulating the local blood flow and the vascular tone, and altogether make up the concept of a “neurovascular unit” (3, 7, 8).

Due to its anatomic location and its unique physiologic properties, BBB disruption is involved in many central nervous system diseases, including epilepsy (1). The involvement of BBB disruption to epileptic seizures has been studied for 3 decades (9–11). Evidence demonstrates that BBB breakdown may induce epileptic seizures, and conversely, seizure-induced BBB disruption may cause further epileptic episodes (12–14). Although the BBB disruption facilitates seizure onset, long-lasting BBB breakdown can result in long-term cognitive impairment which can be observed through altered electroencephalography (EEG) activity (13, 15).

In the past, BBB was considered a barrier that prevented the entrance of antiepileptic drugs to the central nervous system. Nowadays, studies in BBB and epilepsy have been targeting a therapeutic approach to reduce seizure burden (16–19). However, it is also important to understand the dynamics of BBB breakdown during an early event (in the acute phase of epilepsy), which represents a window of opportunity for therapeutic approaches that could prevent the further development of epilepsy. This study was conducted based on the premise that the impairment of brain tissue during the triggering event may determine the organization and functioning of the brain during epileptogenesis, and that the BBB may have a key role in this process (20). Our purpose was to investigate, in male rats, the relationship between pilocarpine-induced status epilepticus (SE) and BBB integrity by determining the time course of the BBB opening and its subsequent recovery during the acute phase of the pilocarpine model.

BBB integrity was assessed by quantitative and morphological methods. We have used sodium fluorescein (NaFl) and Evans blue (EB) vital dyes as markers of the BBB breakdown and analyzed several brain areas during the acute phase of pilocarpine-induced epilepsy. NaFl dye (MW 376 Da) was used as a marker of micromolecule extravasation through the BBB. It does not bind to plasma proteins and is quickly metabolized and excreted, which hinders a lengthy longitudinal study (21). Conversely, EB dye (MW 961 Da) was used as a marker of macromolecular extravasation through the BBB (22), since it binds to albumin serum forming a complex of 68,500 Da that is not excreted. This allows us to perform a longitudinal study and to determine the time course of the BBB breakdown during the acute phase of pilocarpine model of epilepsy.

## Materials and Methods

### Animal Model

Adult male Wistar rats were obtained from the Federal University of Sao Paulo Experimental Models Development Center (CEDEME/Brazil). All experiments were carried out in accordance with the guidelines of the Brazilian National Council for the Control of Animal Experimentation (CONCEA) and approved by the Ethics Committee for Animal Experimentation of the Federal University of Sao Paulo (UNIFESP−01929/08). Room temperature was controlled (22 ± 1°C) and a light-dark cycle was maintained on a 12-h on-off cycle. Food and water were available *ad libitum* throughout the experimental period.

### Study Design

The rats were randomly distributed into two groups: control and pilocarpine-treated. The treated rats received an injection of pilocarpine solution (Pilo; 320–350 mg/Kg *i.p*.) preceded by one dose of methyl-scopolamine 30 min before (1 mg/Kg; *s.c*.) as described by Cavalheiro (23). Rats from the control group were injected with equivalent volumes of saline. For the characterization of the BBB breakdown, different time-points of the pilocarpine model were analyzed: 30 min after pilocarpine injection (group Pilo-30min); and then 1 h (group SE-1h), 5 h (group SE-5h), and 24 h (group SE-24h) after the *status epilepticus* (SE) onset. Additionally, rats from groups SE-5h and SE-24h remained in SE for a period of 3 h, and then the seizures were blocked by diazepam (7–10 mg/Kg *i.p*.).

The Pilo-30 min group was chosen to verify if pilocarpine-induced changes on brain tissue before SE leads to BBB breakdown to micro and macromolecules. The other groups, SE-1h, 5h, and 24h were used to investigate if pilocarpine-induced SE leads to BBB breakdown, and additionally to carry out an investigation about the subsequent closure.

#### Experiment 1: Assessment of BBB Permeability to Micromolecules

NaFl dye (100 mg/Kg) was injected intravenously in the tail vein of the rats (*n* = 4–11 rats/group) 30 min before euthanasia. The rats were then deeply anesthetized intraperitoneally with a mixture of Ketamin (100 mg/Kg) and Xylazine (10 mg/Kg), and perfused transcardially with phosphate buffer saline (PBS; 0.1 M pH 7.4). The brains were quickly removed, and specific regions were dissected: hypothalamus, hippocampus, entorhinal/piriform cortex, and neocortex. Samples were homogenized in cold PBS and proteins were precipitated with trichloroacetic acid at final concentration of 30% (Sigma-Aldrich, St. Louis-Mo-USA), and centrifuged for 10 min at 14,000 g. The supernatants were collected and measured in a spectrophotofluorometer (Spectramax M2; Molecular Devices) at excitation wavelength of 440 nm and emission wavelength of 525 nm (21). The results were expressed in μg of total NaFl obtained from a standard curve and were covariate with the mass of each region.

#### Experiment 2: Assessment of BBB Permeability to Macromolecules

EB dye (80 mg/Kg) was injected intravenously in the tail vein immediately before pilocarpine injection in the group Pilo-30 min, and at the SE onset in rats of groups SE-1h, SE-5h, and SE-24h (*n* = 4–12 rats/group). To assess the integrity of BBB 24 h after SE, an additional group was performed (group SE-24h/EB30min; *n* = 5), in which EB dye was injected 30 min before euthanasia. In the control group, rats received EB dye immediately before saline injection. The brains were quickly removed, photographed for macroscopic analysis, and the specific regions were dissected as described before. The samples were homogenized in cold PBS, protein precipitated with trichloroacetic acid 30% (Sigma-Aldrich, St. Louis-Mo-USA) and then centrifuged for 10 min at 14,000 g. The supernatant was collected and read at 610 nm using a microplate reader (Spectramax M2; Molecular Devices) (24). The results were expressed in μg of total EB obtained from a standard curve and were covariate with the mass of each region.

### Morphological Analyses of EB Dye Distribution in Brain Tissue

Based on the results obtained in the macroscopic and quantitative analysis of EB dye, soon after the first clinical signs of SE, EB dye was injected, and the rats were euthanized after 5 and 24 h of dye circulation. Rats from the groups SE-5h and SE-24h were perfused transcardially with PBS followed by 4% paraformaldehyde in PBS (0.1 M, pH 7.4), cryoprotected in 30% sucrose solution, embedded in O.C.T. Compound (Tissue-Tek; Sakura, Alphen aan den Rijn, the Netherlands) and frozen at −80°C. Coronal slices of 30 μm were obtained by cryostat and mounted in a commercial anti-fading agent (Vectashield with DAPI, Vector Labs, Burlingame, CA, USA). The slides were examined and photographed using an Axio Observer D1 fluorescence microscope (Carl Zeiss; Gottingen, Germany). The images were obtained and merged using the multidimensional acquisition tool of Zeiss AxioVision software. Evans blue dye was observed in red (546 nm excitation filter) and nuclear DAPI staining observed in blue (UV filter).

### Immunostaining

To determine which cells captured the EB dye, coronal brain slices from the SE-24hEB group were washed with TBS (0.02 M Tris-buffered saline, pH 7.4), and blocked with 5% skimmed milk powder and 0.3% Triton X-100 diluted in TBS for 2 h at room temperature (RT). Slices were incubated overnight at 4°C with primary antibodies diluted in TBS containing 5% skimmed milk powder and 0.1% Triton X-100. The antibodies used were: mouse anti-NeuN (1:150; #MAB377–Millipore) for neuron detection, rabbit anti-GFAP (1:150; #G9269–Sigma Aldrich) for astrocyte labeling, goat anti-OLIG-2 (1:150; #AB9610–Millipore) for oligodendrocyte lineage, and goat anti-IBA-1 (1:150; #AB5076–Abcam) for microglia. Slices were then washed with TBS and incubated with fluorophore-labeled secondary antibody (1:200, Alexa Fluor 488; Thermo Fisher Scientific) in TBS with 0.l% Triton X-100 for 2 h at RT, in the dark. Slides were mounted as described above and were examined and photographed with the same fluorescence microscope mentioned. Evans blue dye was observed in red (546 nm emission filter), immunostained cells in green (488 nm excitation filter), and nuclear DAPI in blue (UV filter).

### Statistical Analyses

To verify the Gaussian distribution and homogeneity of data, the Shapiro-Wilk normality test and Levene test were applied. The data were standardized with z-score and the homogeneity submitted to Welch's correction, if necessary. General Linear Model (GLM) using one-way analysis of covariance (ANCOVA), followed by the Sidak *post-hoc* test, was performed for comparison among the groups. A value of *p* < 0.05 indicated statistical significance. The results were expressed as means ± standard error of the mean (SEM). Statistical analyses and graphics were performed using IBM SPSS Statistics 20 and GraphPadPrism 7.0 software.

## Results

### BBB Breakdown to NaFl Dye in the Acute Phase of Epilepsy Is Quick and Time-Dependent

**Figure 1:**
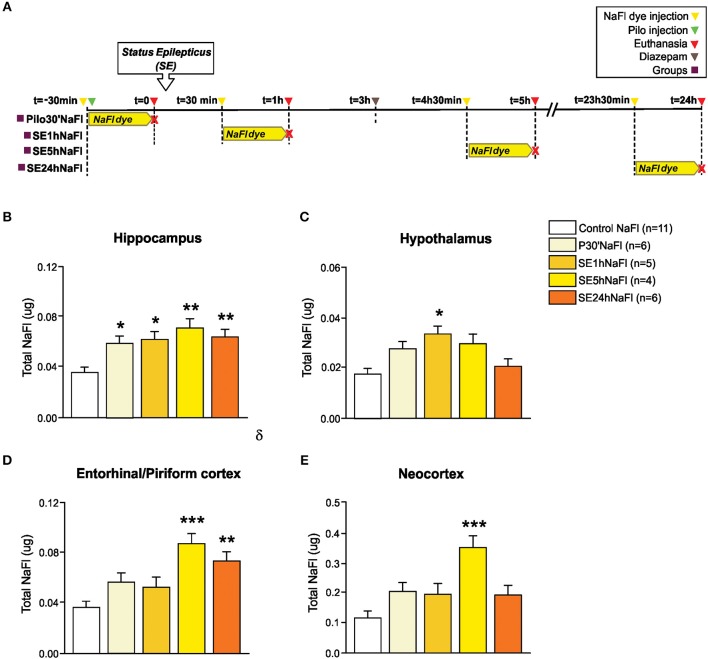
Increased permeability of the BBB to the NaFl dye during the acute phase of the pilocarpine model of epilepsy. In **(A)**, a schematic representation of the experimental design for evaluation of the BBB permeability to micromolecules: in the Pilo30'NaFl group, the NaFl dye was intravenously injected immediately before the pilocarpine (i.p.), and the animals were euthanized 30 min after PILO-injection. In the SE1hNaFl group, the NaFl dye was injected 30 min after the beginning of the *status epilepticus* (SE), and the animals were euthanized 1 h after SE onset. In the SE5hNaFl group, the NaFl dye was injected 4 h 30 min after the beginning of the SE, and the animals euthanized 5 h after SE onset. In the SE24hNaFl group, the NaFl dye was injected at 23 h 30 min after the beginning of the SE, and the animals were euthanized 24 h after SE onset. Note that rats from SE5hNaFl and SE24hNaFl groups had the seizures blocked with diazepam 3 h after SE initiation. In **(B–E)**, quantitative analysis shows that BBB tracer NaFl dye can be detected since the initial periods of the acute phase of the pilocarpine model in the hippocampus **(B)**, hypothalamus **(C)**, entorhinal/piriform cortex **(D)**, and neocortex **(E)**. Data expressed as mean ± SEM. ^*^p < 0.05, ^**^p < 0.01, ^***^p < 0.001 in comparison with control groups by one-way ANCOVA (mass of the region as covariate) and Sidak *post-hoc*.

In *Experiment 1*, we evaluated the permeability of BBB to NaFl dye at different time-points in the acute phase of pilocarpine model of epilepsy. The schematic representation of the experimental design using NaFl dye is depicted in Figure 1A. One-way ANCOVA showed a significant increase in the BBB permeability to NaFl dye in the hippocampus [*F*_(4, 26)_ = 7.101; *p* = 0.001, η^2^ = 0.522], hypothalamus [*F*_(4, 26)_ = 5.922; *p* = 0.002, η^2^^=^ 0.477], entorhinal/piriform cortex [*F*_(4, 26)_ = 7.898; *p* = 0.0001, η^2^ = 0.549], and neocortex [*F*_(4, 26)_ = 7.241; *p* = 0.0001, η^2^ = 0.527]. The mass of each region, used as covariate, was not significant. After Sidak *post-hoc*, our quantitative analysis showed that 30 min after PILO injection (Pilo30'NaFl group), an increased amount of NaFl dye was detected only in the hippocampus (Figure 1B). One hour after SE onset (SE1hNaFl group), a significant increase in NaFl dye persisted in the hippocampus (Figure 1B), and was additionally observed in the hypothalamus (Figure 1C). Five hour after SE began (SE5hNaFl group), the areas with higher NaFl dye were the hippocampus, entorhinal/piriform cortex, and neocortex (Figures 1B,D,E), but not in the hypothalamus (Figure 1C). Twenty-four hour after SE began (SE24hNaFl group), we observed an increase of NaFl dye only in the hippocampus and entorhinal/piriform cortex (Figures 1B,D).

### BBB Breakdown to EB Dye in the Acute Phase of Epilepsy Is Late and Possibly Recoverable

**Figure 2:**
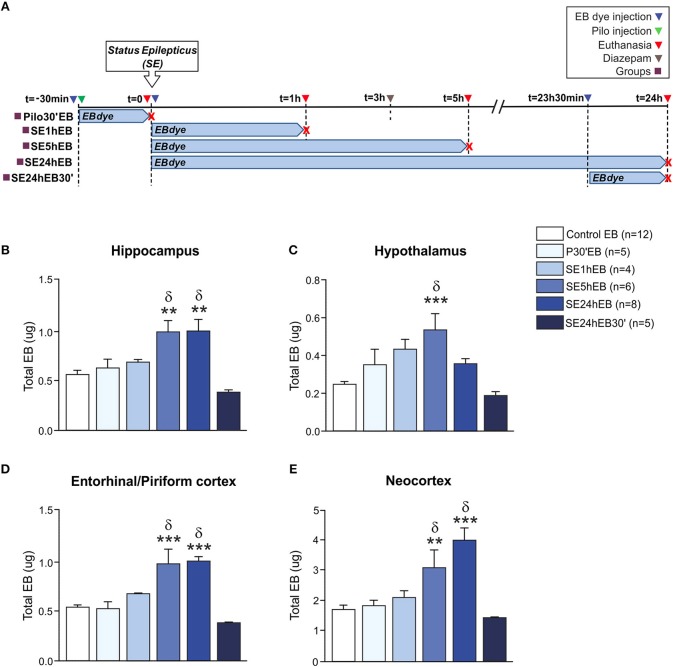
BBB breakdown to the EB dye during the acute phase of the pilocarpine model of epilepsy. In **(A)**, a schematic representation of the experimental design for evaluation of the BBB permeability to macromolecules: in the Pilo30'EB group, Evans blue (EB) dye was intravenously injected immediately before pilocarpine (i.p.), and the animals were euthanized 30 min after PILO injection. In the SE1hEB group, the EB dye was injected at the beginning of the *status epilepticus* (SE), and animals were euthanized 1 h after SE onset. In the SE5hEB group, EB dye was injected at the beginning of the SE, and animals euthanized 5 h after SE onset. In the SE24hEB group, EB dye was injected at the beginning of the SE, and animals were euthanized 24 h after SE onset. In the SE24hEB30' group, EB dye was injected 23 h 30 min after the beginning of the SE, and the animals were euthanized 24 h after SE onset (i.e., EB dye remained in the bloodstream for 30 min). Note that rats from groups SE5hEB, SE24hEB, and SE24hEB30' had seizures blocked with diazepam 3 h after SE initiation. In **(B–E)**, quantitative analysis shows that BBB tracer EB can be detected only between 5 and 24 h after SE beginning in the hippocampus **(B)**, hypothalamus **(C)**, entorhinal/piriform cortex **(D)**, and neocortex **(E)**. Data expressed as mean ± SEM. ^**^p < 0.01, ^***^p < 0.001 in comparison with control groups and ^δ^*p* < 0.001 in comparison with SE24hEB30min by one-way ANCOVA (mass of the region as covariate) and Sidak *post-hoc*.

In *Experiment 2*, we evaluated the BBB permeability to EB dye at the same time-points as the NaFl evaluation. The schematic representation of the experimental design using EB dye is depicted in Figure 2A. One-way ANCOVA showed a significant increase in the BBB permeability to EB dye in the hippocampus [*F*_(5, 33)_ = 7.535; *p* = 0.0001, η^2^ = 0.533], neocortex [*F*_(5, 33)_ = 9.852, *p* = 0.0001, η^2^ = 0.599], hypothalamus [*F*_(5, 33)_ = 5.852; *p* = 0.001, η^2^ = 0.470], and entorhinal/piriform cortex [*F*_(5, 33)_ = 10.819; *p* = 0.0001, η^2^ = 0.621]. The mass of each region, used as covariate, was not significant. After Sidak *post-hoc*, the quantitative analysis carried out 5 h after the SE onset (SE5hEB group) showed a significant increase of EB dye in all brain areas analyzed: hippocampus, hypothalamus, entorhinal/piriform cortex, and neocortex (Figures 2B–E). Twenty-four hour after SE began (SE24hEB group), the areas with increased EB dye concentration were the hippocampus, entorhinal/piriform cortex, and neocortex(Figures 2B,D,E), but not in the hypothalamus (Figure 2C). In the group that received EB dye injection 23h30min after SE began (SE24hEB30' group), we did not observe any increase of EB dye in the regions analyzed.

### BBB Breakdown to EB Dye in the Acute Phase of Epilepsy Can Be Seen Macroscopically

**Figure 3:**
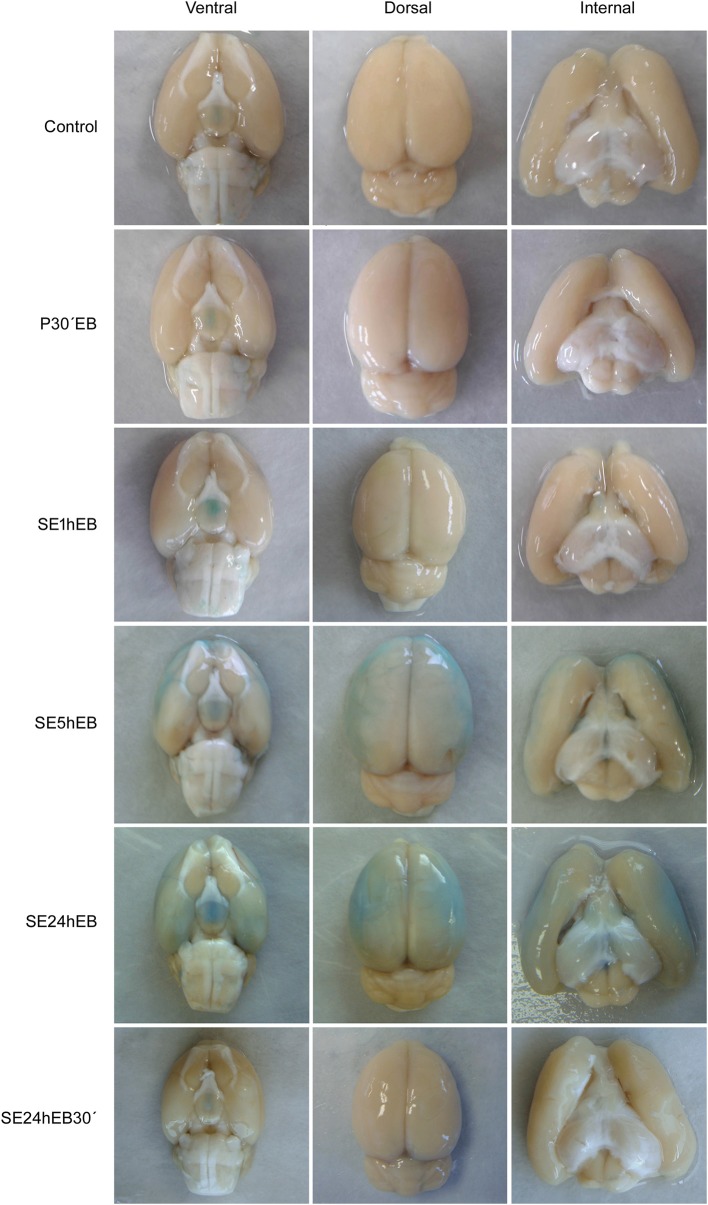
Macroscopic analyses of rats' brains after EB dye injection during the acute phase of the pilocarpine-induced epilepsy model. Representative images of the ventral (left column), dorsal (middle column), and internal (right column) views of the brains after EB injection. Note the EB dye in the neocortex of rats from the SE5hEB group and in the SE24hEB group, the presence of EB dye in the neocortex and entorhinal cortex. In the SE24hEB30' group, note the absence of dye in the brain tissue.

Macroscopic analysis of brains from Control, P30'EB, and SE1hEB groups did not show the presence of EB dye (Figure 3, first three rows). In the SE5hEB group, we observed that some rats showed EB extravasation to the brain tissue (Figure 3, fourth row), whereas in some other animals in the group, this was not observed. In the SE24hEB group, all animals showed diffuse leakage of EB dye to the brain tissue (Figure 3, fifth row). The SE24hEB30' group did not show the presence of EB dye in the brain tissue (Figure 3, bottom row).

### Cells of Brain Tissue Capture EB Dye After BBB Disruption

**Figure 4:**
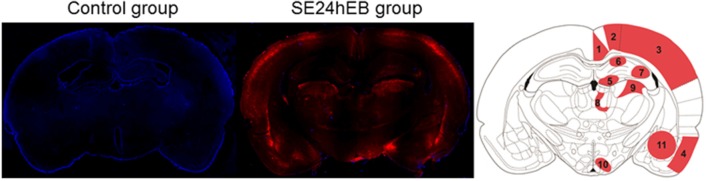
Panoramic view of the brain showing the Evans blue (EB) dye distribution after BBB breakdown. Representative brain slices from Control (left panel) and SE24EB (middle panel) groups obtained by fluorescence microscope. In blue, nuclei of the cells evidenced with DAPI. In red, fluorescence emitted by the EB dye, indicating the sites of BBB breakdown in the SE24hEB group. In the right panel, schematic representation of the main brain areas affected by BBB breakdown in the group SE24hEB: (1) retrosplenial granular cortex (coRSG); (2) motor cortex (coM); (3) somatosensory cortex (coS); (4) piriform cortex (coPir); (5) dentate gyrus (DG); (6) hippocampal CA1 area; (7) hippocampal CA3 area; (8) mediodorsal nucleus of the thalamus; (9) laterodorsal nucleus of the thalamus; (10) ventromedial nucleus of the hypothalamus (VMH); and (11) amygdaloid complex. Bregma−3.14 mm anteroposterior adapted from the rat brain atlas (25). Photomicrographs were taken at a magnification of 100X and panels constructed with Autostitch software (Copyright University of British Columbia).

**Figure 5:**
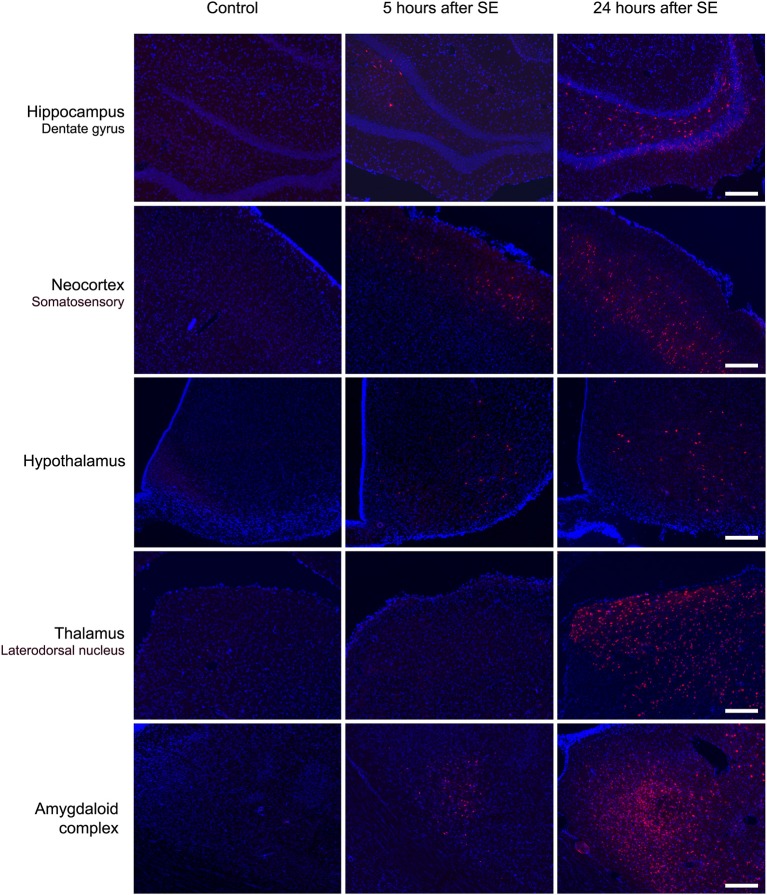
Brain areas affected by BBB breakdown to EB dye in the acute phase of the pilocarpine-induced epilepsy model. Representative photomicrographs obtained under a fluorescence microscope show the main brain areas affected by BBB breakdown: hippocampus, neocortex, hypothalamus, thalamus, and amygdala. Cells containing the Evans blue (EB) fluorescent dye appear as red. Nuclei of the cells evidenced with DAPI are blue. Note that, in the Control group, the cells do not contain EB dye, while in group SE5hEB, there are considerably fewer cells that captured EB than in group SE24hEB, where they are widely distributed into the brain areas analyzed. Magnification: 100X. Bar = 200 μm.

Microscopic analysis of the SE5hEB and SE24hEB groups confirmed a leakage of EB dye in many brain areas when compared to Control group. The EB dye leakage across the disrupted BBB was captured by cells from the neocortex, hippocampus, and hypothalamus, in addition to the thalamus and amygdala cells (Figures 4, 5, and Supplementary Figure 1).

### Neurons, Astrocytes, and Oligodendrocytes Capture EB Dye After BBB Disruption

**Figure 6:**
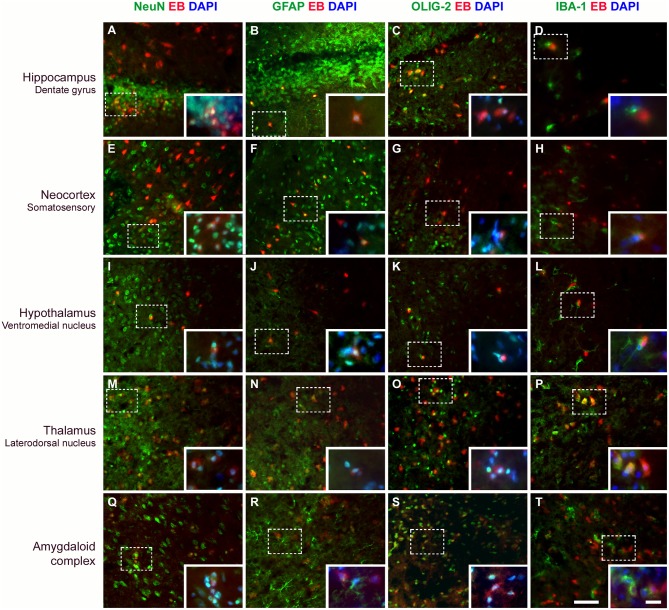
Evans blue (EB) dye is captured by different cell types after BBB breakdown. Immunostaining for detection of neurons (NeuN), astrocytes (GFAP), oligodendrocytes (OLIG-2), and microglia (IBA-1) in various brain areas, 24 h after SE. Positive cells are shown in green. After BBB breakdown, the complex albumin-EB dye enters the brain and is captured by damaged cells (shown in red). Double-staining for the cell types and the EB dye shows that they are mainly neurons, astrocytes, and oligodendrocytes (see in yellow, as a result of red and green fluorescence overlap; larger images). *Inserts* show higher magnification of affected cells (dotted rectangle), with nuclei evidenced with DAPI (shown in blue). White color corresponds to the green, red, and blue fluorescence overlap. The first column of images shows that neurons containing EB are observed in all analyzed areas **(A,E,I,M,Q)**. In the second column, astrocytes are found containing EB **(B,F,J,N)** or involving cells that captured the dye (see amygdaloid complex; **R**). In the third column, oligodendrocytes that captured EB are also observed in all brain areas **(C,G,O,S)**, except in the hypothalamus **(K)**. The fourth column shows that microglia exhibiting thicker branches projections suggestive of activated cells are found involving the EB-containing cells **(D,H,L,T)**, except in the thalamus where macrophage-shaped microglia, are seen containing the dye **(P)**. Magnification: 400X (larger images) and 630X (inserts). Bars = 20 μm (larger images) and 10 μm (inserts).

Immunostaining of the brain tissue performed 24 h after SE (SE24hEB group) revealed that cells that captured EB are mainly neurons, astrocytes, and oligodendrocytes. Microglia exhibiting thicker branches suggestive of activated cells were found mainly involving the EB-containing cells, although macrophage-shaped microglia that captured the dye were also found. Detailed photographic documentation of the various brain areas affected are shown in Figure 6 and Supplementary Figure 2.

## Discussion

Several exogenous and fluorescent BBB tracers have been used for functional and structural analysis of BBB permeability (26). In our experiments, for evaluating the permeability of BBB to micromolecules during the acute phase of the pilocarpine model, we used NaFl dye as a tracer and observed an increased BBB permeability in the hippocampus, that occurred up to 30 min after pilocarpine injection. BBB permeability to micromolecules in the hypothalamus increased only 1 h after SE onset while in the entorhinal/piriform cortex and in the neocortex it was observed 5 h after SE began. Particularly in the hippocampus and entorhinal/piriform cortex, the BBB leakage of micromolecules persisted until the end of the experimental period (24 h after SE onset). Similar results were found by van Vliet et al. (20), who demonstrated, through MRI and microscopic analysis using fluorescein tracer, BBB leakage 1 day after kainic acid-induced SE in Sprague–Dawley rats. Our results show that increased BBB permeability to micromolecules is a dynamic phenomenon, and that, mostly in the hypothalamus, an adaptive property of BBB can be observed, preventing the breakdown to macromolecules which would cause subsequent brain damage.

Regardless of etiology, BBB disruption has been shown to be associated with epileptogenesis after injury (27, 28) both clinically (29) and experimentally (12, 30, 31). In this context, the early adaptive behavior of BBB damage is important especially because the chemical microenvironment of the synapses and the brain extracellular space play a key role in the generation of seizures (17, 32, 33).

There is evidence that the initial episode, i.e., status epilepticus, can be induced by pilocarpine through a primary peripheral effect on white blood cells, leading to increased serum levels of IL-1β, which alters BBB permeability (34). BBB leakage triggers a complex cascade which includes protein extravasation, impaired potassium buffering, brain inflammation, angiogenesis, and changes in multidrug transporters and metabolic enzymes (17). The ionic imbalance due to potassium accumulation in the extracellular space, induced by BBB disruption, facilitates convulsant activity by stimulation of muscarinic receptors in the brain, leading to neuronal hyperexcitability (35). This has also been demonstrated by electrophysiological investigations performed in cultured endothelium and brain slices in which, even in the presence of cholinergic agonists at high concentrations, the epileptiform activities only occurred in the presence of elevated level of potassium in the extracellular environment (30). Taken together, these findings confirm the hypothesis that BBB disruption during the acute phase of the pilocarpine model of epilepsy may contribute to the triggering and propagation of electrical discharges, and subsequently lead to nervous tissue damage.

Besides increased BBB permeability to micromolecules, our results also demonstrate the existence of a temporal window of BBB breakdown to macromolecules in the acute phase of pilocarpine-induced epilepsy. Our macroscopic and quantitative findings show that while some animals, which were all euthanized 5 h after the commencement of SE, had already had extravasation of EB dye; others had not. This variability among animals could be due to different time-dependent responses to the pilocarpine-induced SE, particularly, individual differences in the oxidative stress and neuroinflammation (36, 37), and indicates that BBB breakdown to macromolecules occurs around 5 h after SE onset. Conversely, 24 h after SE onset, all animals showed EB dye in the brain tissue. Results show that even though the BBB function seems to have been restored 24 h after SE started, the consequences of the macromolecules extravasation into the brain tissue, that had occurred earlier, were widely spread in the brain. The animals from SE-24h group, which received EB dye at SE onset and were euthanized 24 h later, showed a significant increase in BBB breakdown and EB dye extravasation in several brain areas (hippocampus, entorhinal cortex, and neocortex). It is noteworthy that animals which received EB dye 23 h 30 min after SE started did not show EB dye extravasation to the nervous tissue. Taken together, this data indicates that during the period between 5 and 24 h after SE onset, there is a BBB breakdown to macromolecules and restoration. Unfortunately, the extensive gap between our experimental periods does not allow us to identify the moment when the BBB restoration happened. Future studies should be conducted in order to elucidate it.

In agreement with our findings, Marchi et al. (30) demonstrated, by histological assays, points of leakage of fluorescein isothiocyanate (FITC)-labeled albumin in the limbic system of rats during the pilocarpine-induced SE. The authors attribute the onset of seizures to increased BBB permeability in areas sensitive to cholinergic agents. Moreover, our results are also consistent with others who reported that, after pilocarpine-induced SE, these brain areas display the greatest inflammatory response in positron emission tomography (PET) (38, 39).

Some studies have emphasized the potential involvement of a primary lesion of the BBB in the epileptogenesis by exposing the brain parenchyma to serum albumin (27, 40–42). Here we showed that, after BBB breakdown, the EB-bound albumin enters the brain and is captured mainly by neurons, astrocytes, and oligodendrocytes. These findings are in accordance with literature that has demonstrated that not only neurons, but also astrocytes and microglia can uptake albumin after SE (43–45). In microglia, albumin can be found as a result of a phagocytic activity that protects the brain during SE (12). However, our data show that EB dye was captured mainly by damaged cells, and less by reactive microglia, which was chemoattracted to the injured cells, wrapping them. This can be explained by the fact that, when activated, microglial cells release cytokines, chemokines, growth factors, and other molecules to attenuate or prevent brain damage, besides removing debris by phagocytosis (46). Astrocytes are particularly sensitive to SE-induced brain tissue damage. These cells can uptake albumin through TGF-β receptors, leading to increased intracellular calcium that results in downregulation of inward rectifying potassium (Kir 4.1) and aquaporin-4 (AQP4) channels (47–49), reducing the buffer of extracellular potassium and facilitating NMDA-mediated neuronal hyperexcitability and epileptiform activity (33, 44, 50, 51). Although the presence of albumin in neurons can lead to death (12), it seems that albumin *per se* is not neurotoxic; it can only be captured by damaged neurons (44). In fact, when EB dye is used to evaluate the BBB integrity, it strongly binds to albumin in the bloodstream and forms a complex with high molecular weight. In the case of BBB breakdown, this complex enters the brain and is captured only by cells that have lost their selective permeability of the membrane, but not by cells with an intact membrane (52). Consequently, although using 30 μm thick sections, we were able to detect the distribution of damaged cells after BBB disruption, and then confirm that all cell types are affected during the acute phase of pilocarpine model, with preference for neurons, astrocytes, and oligodendrocytes.

Although the exact moment of BBB opening was not precisely determined, research into pilocarpine-induced SE has already included testing of some new therapies for the prevention of BBB disruption. Fu et al. (53) hypothesized that HMGB1 (high mobility group box-1), a pro-inflammatory cytokine-like molecule, may be involved in the development of epileptogenesis in mice through BBB disruption and induction of inflammatory processes. These authors showed that treatment with anti-HMGB1 decreases the concentration of EB dye in the thalamus and hypothalamus during pilocarpine-induced SE, while the control group did not show any reduction in BBB leakage. On the other hand, injection of recombinant human HMGB1 after pilocarpine, enhanced the leakage of EB dye in an HMGB1 dose-dependent manner. However, the authors injected EB dye 2 h after pilocarpine administration and then waited for only 2 h for the animals' euthanasia. Although they showed the efficacy of the therapy with anti-HMGB1 in preventing BBB disruption, some benefits may have been overlooked due to a lack of knowledge about the time-point of the BBB breakdown in the pilocarpine model. They observed EB dye extravasation only in the thalamus and hypothalamus possibly because, 4 h after pilocarpine injection, the BBB in other areas is still preserved. Although the anti-HMGB1 treatment has been quite successful in epileptogenesis (54), it still needs to be tested after longer periods of SE, and other brain areas should be analyzed.

In recent years, some efforts have been concentrated to develop a quantitative assessment of vascular injury, which could be an important diagnostic, predictive, and pharmacodynamic biomarker for epilepsy. Recently, Bar-Klei et al. (55) proposed a feasible contrast-enhanced modality to detect regions with BBB hyper-permeability in humans throughout two types of T1-weighted MRI sequences. However, further studies are still required to confirm the most sensitive and reliable protocol for BBB imaging, which, in the coming years, will probably be a big step forward for clinical practice.

**Figure 7:**
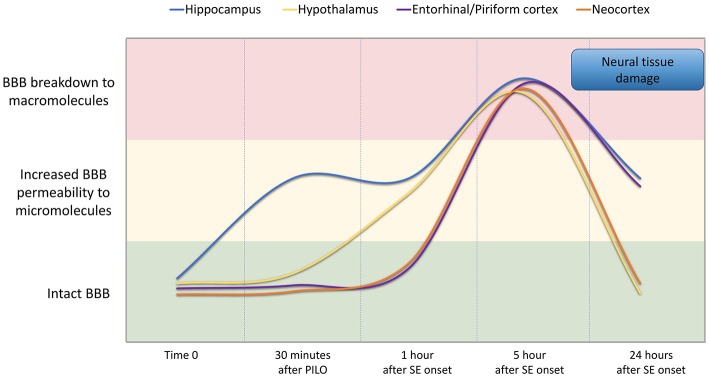
Representation of BBB permeability during the acute phase of the pilocarpine model. The colored bands correspond to the BBB status: intact (green band), increased permeability to micromolecules (yellow band), and breakdown to macromolecules (red band). The lines represent the dynamics of the BBB opening and restoration over time in the regions of the hippocampus (blue line), hypothalamus (yellow line), entorhinal and pyriform cortex (purple line), and neocortex (orange line). Note that increased BBB permeability for micromolecules is observed from 30 min after PILO injection, and the BBB breakdown for macromolecules occurs about 5 h after SE onset. Although the BBB permeability to macromolecules is restored 24 h after SE initiation, the leakage of micromolecules persists and the consequences of BBB degradation on brain tissue are widely disseminated in the brain.

In conclusion, our findings indicate that BBB breakdown is a dynamic phenomenon and time-dependent, i.e., it happens at specific time-points of the acute phase of pilocarpine model of epilepsy, recovering in part its integrity afterwards (Figure 7). We show that pilocarpine-induced changes on brain tissue initially increased the permeability of the BBB to micromolecules, and subsequently, after SE, the BBB breakdown to macromolecules occurred. Although the BBB permeability to macromolecules is restored 24 h after SE, the leakage of micromolecules persists and the consequences of BBB degradation are widely disseminated in the brain, which in turn may induce further episodes of BBB breakdown. Together, our data reveal the existence of a temporal window of BBB dysfunction during the acute phase of the pilocarpine model that is important for the development of therapeutic strategies to prevent the epileptogenesis.

## Ethics Statement

Adult male Wistar rats were obtained from the Federal University of São Paulo Experimental Models Development Center (CEDEME/Brazil). All experiments were carried out in accordance with the guidelines of the Brazilian National Council for the Control of Animal Experimentation (CONCEA) and approved by the Ethics Committee for Animal Experimentation of the Federal University of São Paulo (UNIFESP−01929/08).

## Author Contributions

NM, EC, PT, JM, MO, GM, and LL carried out the experiments. AP, JC, and AdS contributed to performing the experiments and in the data interpretation. LL designed the study and performed the statistical analyses. NM, GM, and LL interpreted the results and wrote the manuscript. All authors have approved the final manuscript as submitted.

### Conflict of Interest Statement

The authors declare that the research was conducted in the absence of any commercial or financial relationships that could be construed as a potential conflict of interest.

## Supplementary Material

Figure S1 Brain areas affected by BBB breakdown to EB dye in the acute phase of the pilocarpine-induced epilepsy model. Representative photomicrographs obtained under a fluorescence microscope show the main brain areas affected by BBB breakdown: hippocampus, neocortex, hypothalamus, thalamus, and amygdala. Cells containing the Evans blue (EB) fluorescent dye appear as red. Nuclei of the cells evidenced with DAPI are blue. Note that, in the Control group, the cells do not contain EB dye, while in group SE5hEB, there are considerably fewer cells that captured EB than in group SE24hEB, where they are widely distributed into the brain areas analyzed. Magnification: 100X. Bar = 200 μm.

Figure S2 Evans blue (EB) dye is captured by different cell types after BBB breakdown. Immunostaining for detection of neurons (NeuN), astrocytes (GFAP), oligodendrocytes (OLIG-2), and microglia (IBA-1) in various brain areas, 24 h after SE. Positive cells are shown in green. After BBB breakdown, the complex albumin-EB dye enters the brain and is captured by damaged cells (shown in red). Double-staining for the cell types and the EB dye shows that they are mainly neurons, astrocytes, and oligodendrocytes (see in yellow, as a result of red and green fluorescence overlap; larger images). *Inserts* show higher magnification of affected cells (dotted rectangle), with nuclei evidenced with DAPI (shown in blue). White color corresponds to the green, red, and blue fluorescence overlap. The first column of images shows that neurons containing EB are observed in all analyzed areas **(E,I,M,Q,U)**, except in the CA3 **(A)**. In the second column, astrocytes are found containing EB **(B,F,J,R,V)** or involving cells that captured the dye (see Piriform cortex; **N**). In the third column, oligodendrocytes that captured EB are also observed in all brain areas **(C,G,K,O,S,W)**. The fourth column shows microglia exhibiting thicker branches projections suggestive of activated cells involving the EB-containing cells in all analyzed areas **(D,H,L,P,T,X)**. Magnification: 400X (larger images) and 630X (inserts). Bars = 20 μm (larger images) and 10 μm (inserts).
