# Corrigendum: The Blood-Brain Barrier Breakdown During Acute Phase of the Pilocarpine Model of Epilepsy Is Dynamic and Time-Dependent

**DOI:** 10.3389/fneur.2019.00603

**Published:** 2019-06-10

**Authors:** Natália Ferreira Mendes, Aline Priscila Pansani, Elis Regina Ferreira Carmanhães, Poliana Tange, Juliana Vieira Meireles, Mayara Ochikubo, Jair Ribeiro Chagas, Alexandre Valotta da Silva, Glaucia Monteiro de Castro, Luciana Le Sueur-Maluf

**Affiliations:** ^1^Departamento de Biociências, Universidade Federal de São Paulo, Santos, Brazil; ^2^Departamento de Neurologia e Neurocirurgia, Universidade Federal de São Paulo, São Paulo, Brazil; ^3^Departamento de Psicobiologia, Universidade Federal de São Paulo, São Paulo, Brazil

**Keywords:** epilepsy, blood-brain barrier, pilocarpine, status epilepticus, Evans blue, sodium fluorescein

In the original article, there was a mistake. In the **Discussion**, “Figure 5” was referenced instead of “Figure 7.”

A correction has been made to the **Discussion**, paragraph nine:

“In conclusion, our findings indicate that BBB breakdown is a dynamic phenomenon and time-dependent, i.e., it happens at specific time-points of the acute phase of pilocarpine model of epilepsy, recovering in part its integrity afterwards (Figure 7). We show that pilocarpine-induced changes on brain tissue initially increased the permeability of the BBB to micromolecules, and subsequently, after SE, the BBB breakdown to macromolecules occurred. Although the BBB permeability to macromolecules is restored 24 h after SE, the leakage of micromolecules persists and the consequences of BBB degradation are widely disseminated in the brain, which in turn may induce further episodes of BBB breakdown. Together, our data reveal the existence of a temporal window of BBB dysfunction during the acute phase of the pilocarpine model that is important for the development of therapeutic strategies to prevent the epileptogenesis.”

Additionally, Figure 7 was not provided in the original manuscript. Figure 7 appears below.

**Figure 7:**
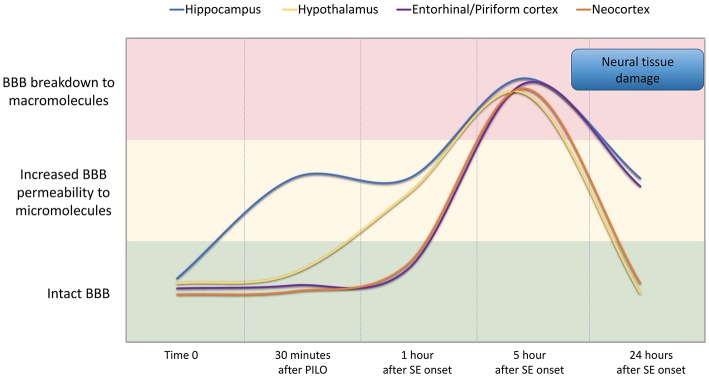
Representation of BBB permeability during the acute phase of the pilocarpine model. The colored bands correspond to the BBB status: intact (green band), increased permeability to micromolecules (yellow band), and breakdown to macromolecules (red band). The lines represent the dynamics of the BBB opening and restoration over time in the regions of the hippocampus (blue line), hypothalamus (yellow line), entorhinal and pyriform cortex (purple line), and neocortex (orange line). Note that increased BBB permeability for micromolecules is observed from 30 min after PILO injection, and the BBB breakdown for macromolecules occurs about 5 h after SE onset. Although the BBB permeability to macromolecules is restored 24 h after SE initiation, the leakage of micromolecules persists and the consequences of BBB degradation on brain tissue are widely disseminated in the brain.

The authors apologize for this error and state that this does not change the scientific conclusions of the article in any way. The original article has been updated.

