# Supplementary figures and images for: The Blood-Brain Barrier Breakdown During Acute Phase of the Pilocarpine Model of Epilepsy Is Dynamic and Time-Dependent
Source: Front Neurol. 2019 Apr 16;10:382. doi: 10.3389/fneur.2019.00382 (PMC6477033; doi:10.3389/fneur.2019.00382)

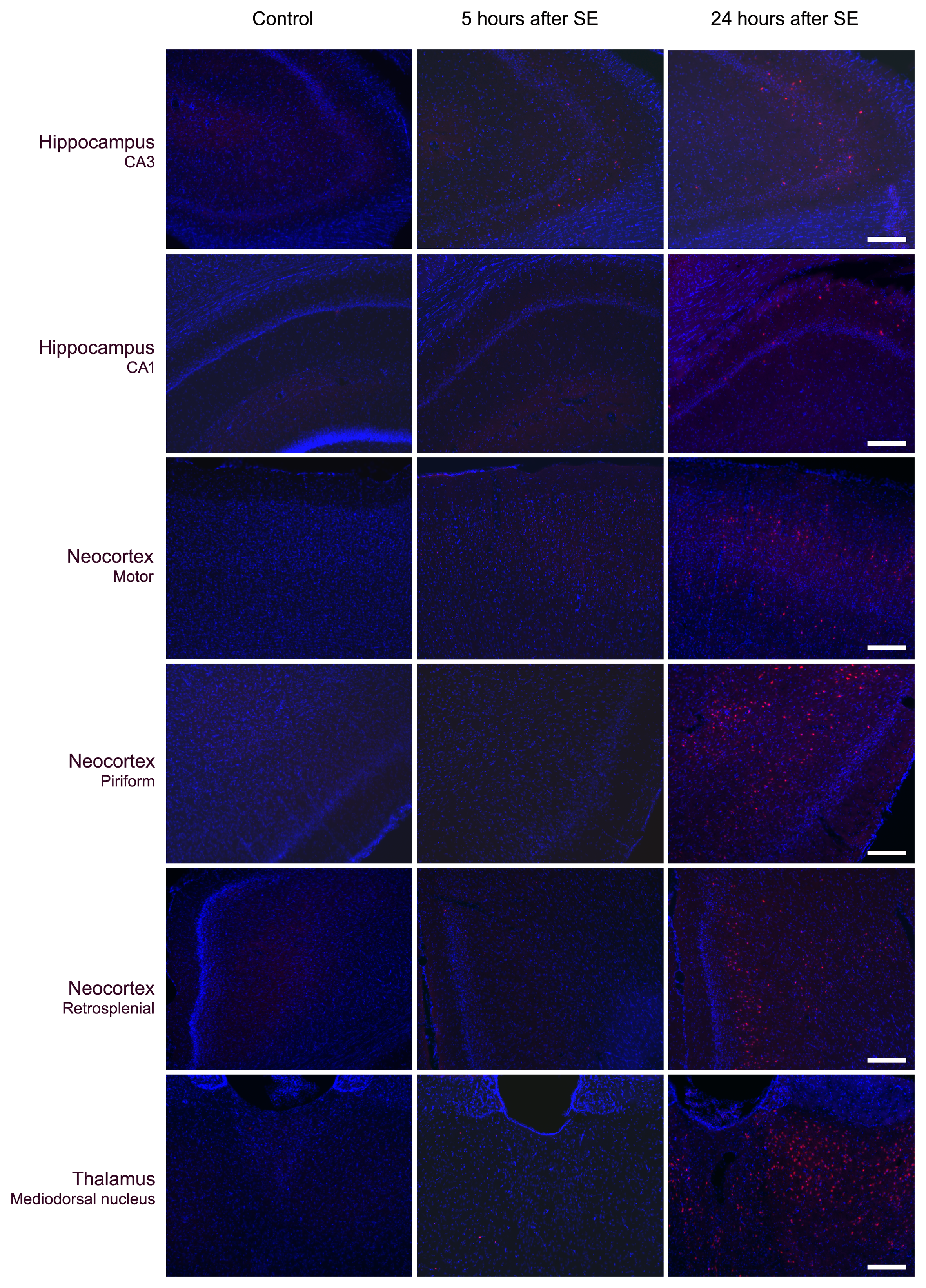

Supplement: Figure S1 — Brain areas affected by BBB breakdown to EB dye in the acute phase of the pilocarpine-induced epilepsy model. Representative photomicrographs obtained under a fluorescence microscope show the main brain areas affected by BBB breakdown: hippocampus, neocortex, hypothalamus, thalamus, and amygdala. Cells containing the Evans blue (EB) fluorescent dye appear as red. Nuclei of the cells evidenced with DAPI are blue. Note that, in the Control group, the cells do not contain EB dye, while in group SE5hEB, there are considerably fewer cells that captured EB than in group SE24hEB, where they are widely distributed into the brain areas analyzed. Magnification: 100X. Bar = 200 μm. [file Image_1.TIF]

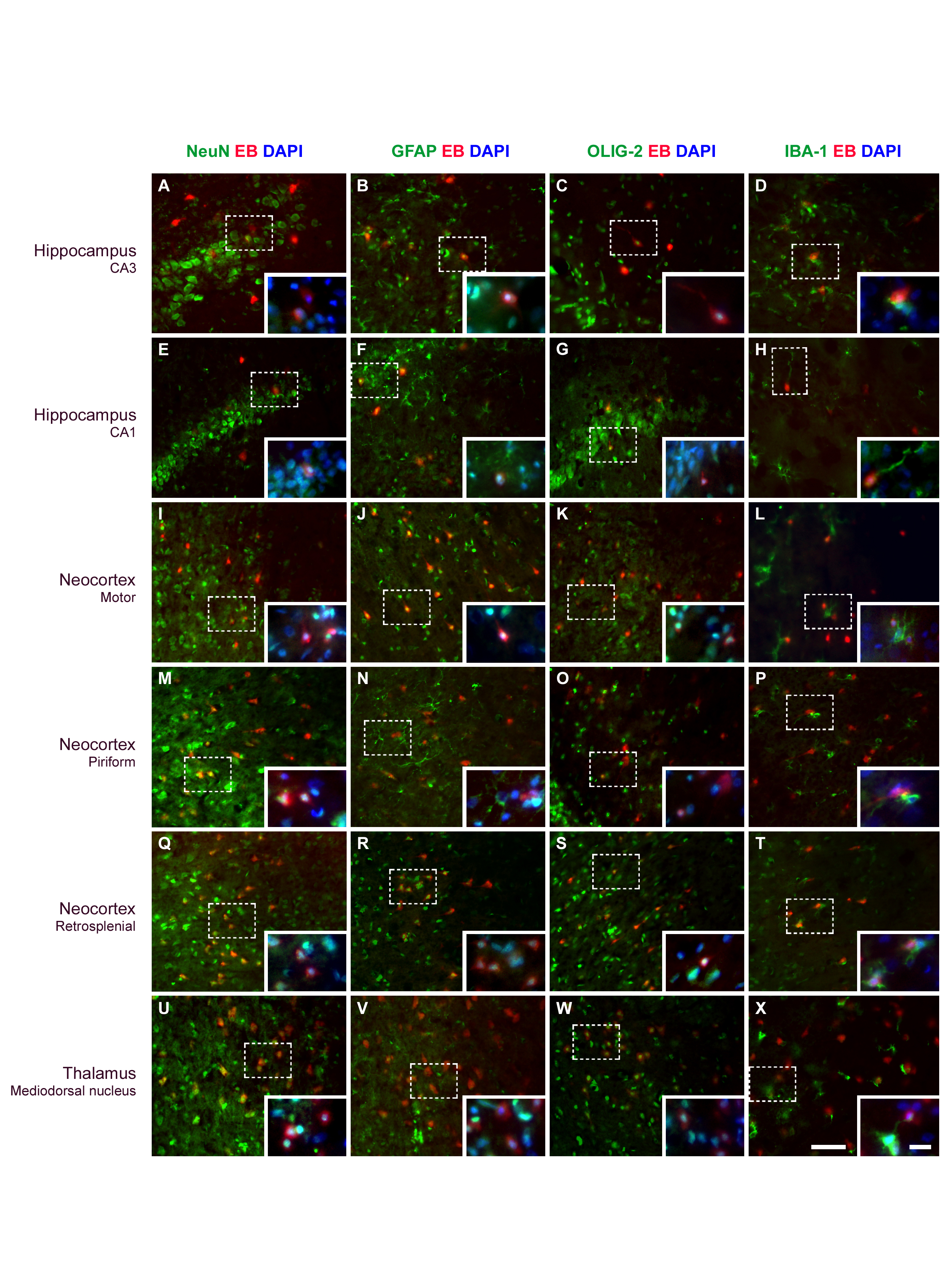

Supplement: Figure S2 — Evans blue (EB) dye is captured by different cell types after BBB breakdown. Immunostaining for detection of neurons (NeuN), astrocytes (GFAP), oligodendrocytes (OLIG-2), and microglia (IBA-1) in various brain areas, 24 h after SE. Positive cells are shown in green. After BBB breakdown, the complex albumin-EB dye enters the brain and is captured by damaged cells (shown in red). Double-staining for the cell types and the EB dye shows that they are mainly neurons, astrocytes, and oligodendrocytes (see in yellow, as a result of red and green fluorescence overlap; larger images). Inserts show higher magnification of affected cells (dotted rectangle), with nuclei evidenced with DAPI (shown in blue). White color corresponds to the green, red, and blue fluorescence overlap. The first column of images shows that neurons containing EB are observed in all analyzed areas (E,I,M,Q,U), except in the CA3 (A). In the second column, astrocytes are found containing EB (B,F,J,R,V) or involving cells that captured the dye (see Piriform cortex; N). In the third column, oligodendrocytes that captured EB are also observed in all brain areas (C,G,K,O,S,W). The fourth column shows microglia exhibiting thicker branches projections suggestive of activated cells involving the EB-containing cells in all analyzed areas (D,H,L,P,T,X). Magnification: 400X (larger images) and 630X (inserts). Bars = 20 μm (larger images) and 10 μm (inserts). [file Image_2.jpg]
